# When Assessing Intra-Familial Relationships, Are Sociologists, Psychoanalysts and Psychiatrists Really Considering Different Constructs? An Empirical Study

**DOI:** 10.1371/journal.pone.0132153

**Published:** 2015-07-17

**Authors:** Bruno Falissard, Caroline Barry, Christine Hassler, Muriel Letrait, Guillaume Macher, François Marty, Elsa Ramos, Anne Revah-Lévy, Philippe Robert, François de Singly

**Affiliations:** 1 INSERM U669, Université Paris-Sud and Université Paris-Descartes, Paris, France; 2 CERLIS, Université Paris-Descartes, Paris, France; 3 EA 4046, Université Paris-Descartes, Paris, France; University of Western Brittany, FRANCE

## Abstract

This paper aimed to look for the existence of a common core when envisaging intra-familial interactions as perceived by adolescents, which could be shared by sociology, psychoanalysis and child and adolescent psychiatry. An empirical study based on a mixed-method design collected the responses of 194 adolescents to the instruction “In the next half hour, would you please write as freely as you wish about your relationships in your family, explaining how things are”. All answers were then analyzed and 18 dimensions related to 3 different theoretical frameworks were rated blind using numerical scores by two independent raters from each discipline. Inter-rater reliability was good. A parallel analysis evidenced a strong underlying factor explaining a large amount of variance (>50%). This factor is bipolar, it reflects the level of positivity/negativity in the adolescent’s point of view concerning his/her intra-familial relationships. A second factor can marginally be considered (10% of the variance). The 2-factor analysis found one factor related to positive feelings and the other to negative feelings. This finding of unidimensionality supports family study as an intervention science.

## Introduction

The field of family assessment measures is very active. As early as 1996, more than 1000 instruments were available in this area [1]. This plethora can be explained by the large number of facets of family functioning and by the fact that family issues interest many disciplines, like psychology, sociology, medicine or educational science. This plethora also points to the fact that we are at the moment far from having a consensual unified theory of the family [2]. Some authors acknowledge positive aspects of this situation—“Growing diversity may be viewed as a sign of health”, but others underline the potential drawbacks “[it] slows the advancement of family psychology as an intervention science” [3].

Even when considering the particular situation of families with an adolescent, the theoretical positions are, indeed, numerous. Gavazzi [4] points to five of them among the most well-known:

1. Family development theory (which focuses on the typical changes experienced by families as they move through their life course, such as marriage, arrival of young children, the moment when the children leave home, and the empty nest);
2. Family system theory (which suggests that families are systems of interconnected subjects, none of whom can be understood in isolation from the whole);
3. Ecological theory (families are an ecosystem, families interact with their environment to form a bigger ecosystem, etc.);
4. Attachment theory (children look for a caregiver when they are distressed, in the hope that they will receive protection and emotional support);
5. Social learning theory (which suggests that children adopt behaviors by the observation of their environment, in particular their family).

In addition to this range of theoretical standpoints, there is also an impressive diversity in methodological, epistemological and even ontological positions implicitly adopted in the research work carried out in the field. Research can use qualitative or quantitative methods, post-positivist or constructivist ontologies. The researcher can claim a neutral position regarding the families studied or, on the contrary, conduct his/her analyses through the lens of his/her own subjectivity.

Ultimately a critical question inevitably arises: in view of this heterogeneity, is communication possible across the field of family studies? This question became a real practical issue in 2008 when the Université Paris-Descartes called for interdisciplinary work on the subject of adolescence. Three research units decided to respond together to the proposal: one team of sociologists (CERLIS: “Research on social bonds”), one team of psychoanalysts (EA4046: “Clinical psychology, psychopathology and psychoanalysis”) and one team of child and adolescent psychiatrists (INSERM U669: “Mental health and public health”). Because of a common interest in family studies, the question of “intra-familial interactions as perceived by adolescents” was chosen as a background for the upcoming research. After a few meetings, it turned out however that it was really difficult to design a common project because of serious methodological and epistemological differences. It was then decided to deal specifically with these differences. Our research question was thus: “beyond our specificities concerning concepts, words, methods and traditions relating to what we call ‘families’, is there a common core, a common perspective that can emerge to address intra-familial interactions as perceived by adolescents?”

Because some researchers in our group favoured qualitative approaches and others quantitative approaches, a mixed method design was adopted:

First, we decided on the data needed to address our research question, and it was agreed a priori that the data would be of a qualitative nature.
Second, on the basis of three explicit theoretical frameworks and of data available from a pilot study, each team (sociologists, psychoanalysts, child and adolescent psychiatrists) conceived a series of dimensions that could be assessed from a textual corpus collected from a sample of adolescents.
Third, multidimensional statistical analyses were used to test whether or not these dimensions had something in common. Some (qualitative) content analyses were also performed, but they are intended to be presented in another paper.

Let us now see this methodology in greater detail.

## Method

### First Phase: Contact and Outline of the Study

Over 18 months the team, composed of 4 sociologists (open to both qualitative and quantitative approaches), 2 psychoanalysts (working in a qualitative perspective) and 2 child and adolescent psychiatrists (one epidemiologist and the other specialized in qualitative methods) met every month. The objective was to share conceptual perspectives and to progressively draft the outline of a study design.

As mentioned above, due to converging interests, it was progressively decided that the study would focus on the adolescent perspective on intra-familial relationships. This option enables a critical moment in the construction of the representations of intra-familial relationships to be approached [5]. Moreover, since it focuses on a single member of the family, the study is easier to implement in practice.

To obtain a good compromise between wealth of content and feasibility, it was decided that the data to be analyzed would consist in the corpus obtained from the following instruction:

“In the next half hour, would you please write as freely as you wish about your relationships in your family, explaining how things are. All that you write is anonymous and no parent or person from your school will read it.”

Basically, this instruction was intended for the setting of a classroom of students from the 6^th^ to the 12^th^ grade according to the American system (roughly from 11 to 18 years old).

### Second Phase: Pilot Study

To determine what information would generally be derived from the answer given by an adolescent to the instruction detailed above, it was decided to conduct a pilot study. Eighty-four adolescents where thus interviewed during school time. 28 adolescents were in the 6^th^ grade and 56 in the 4^th^ grade.

### Third Phase: Operationalization of the Process of Corpus Reduction

From the corpus obtained in the pilot study (the average length of the answers was 224 words), the members of each discipline determined which aspects of their theoretical framework were regularly addressed by the adolescents and which dimensions could thus be explored and rated. These theoretical frameworks and the corresponding dimensions are briefly detailed below.

#### Sociological perspective

According to François de Singly, the modern family has been transformed by a process of individuation of each member in the group [6,7]. In this perspective, the family relationship can be overall positive if it enables each of its members (and here the adolescent in particular) to be recognised as a person on the one hand, and assisted and supported in the construction of his or her identity on the other. Peter Berger and Hans Kellner, following on from George Mead, consider that the parents are "significant others" who take on this role of validating the self more or less adequately [8,9]. For this research a parallel was drawn, from a sociological viewpoint, between this theory of the significant other in the family and the theory of recognition, formulated by Axel Honneth [10]. This theory proposes three types of recognition: loving, legal and social. Roughly speaking, love and affection, personal rights and recognition of merit underpin the construction of the self.

The sociology team operationalized these three types of recognition so as to apprehended the ways in which the adolescent can feel he or she is recognised. Indeed, according to Honneth, being recognised is above all experienced by way of its opposite, contempt. Ideally, recognition of the adolescent is expressed through the love of his or her parents [a], through the recognition of certain rights [b], and the recognition of his/her personal value [c]. These three dimensions were converted into three indicators making up the sociological evaluation grid for the material collected for the study. Two further indicators were added, the relevance of which was obvious in adolescent narratives collected in the pilot study, also relating to recognition: the presence of conflict [d] and the sense of injustice [e]. Indeed, for the adolescent, being recognised implies being treated fairly, that is to say in the same way as his/her siblings, and in a manner appropriate to his/her age. It also implies not being frustrated and bullied.

Finally, in the perspective of the individuation process backed up by the three types of recognition, we set out to assess the degree of differentiation between "me" and "us" within the family, using the following indicator: does the adolescent talk about his/her parents as a whole or group, or as separate individuals with their particular characteristics, whether positive or negative [f]? Thus from a sociological viewpoint we are dealing with indicators relating both to the estimation by the adolescent (subjective viewpoint coherent with individuation) of the recognition he or she has or does not have from parents and siblings, and to his/her view of the more or less individualised functioning of the family.

In all, the six questions relating to the sociological domain are as follows:

- Does the adolescent describe a good affective family atmosphere?
- Does the adolescent feel that his/her parents or step-parents accept the fact that he may want to do some things he/she feels are important?
- Does the adolescent feel that his/her parents or step-parents recognise his/her qualities, approve of his/her tastes and/or valorise his/her abilities?
- Does the adolescent feel he/she is in conflict with at least one parent (or step-parent)?
- Does the adolescent feel he/she is treated unfairly by at least one parent (or step-parent)?
- Does the adolescent talk about his/her parents or step-parents as being part of a whole, or does he/she talk of them as distinct individuals with their particular characteristics, whether positive or negative?

For each aspect, the rater is asked to indicate whether or not the adolescent's answer provides information about the domain in question. If it does, the rater is then required to allocate a score from 0 to 10 (whole numbers) reflecting the level of agreement between the situation described by the adolescent and the content of the item.

#### Psychoanalytical perspective

For Freud, whose theory centres on intra-psychic life, the family was not an object of study in itself. There was for a long period reluctance on the part of psychoanalysts towards family therapies, since they involved the particular risk of giving too much importance—or even exclusive importance—to external objects and to the family history in its potentially objective reality. However, if we look for a definition of the family in terms of its structure, laws, rule and myths, dialogue with other disciplines is not only possible, it is also desirable. The aspect that pinpoints the specificity of the psychoanalytic approach relates to the economic dimension, to traumatic issues, and to group defence mechanisms driven by unconscious alliances that make up the ties among members of a group, as well as between generations.

The "envelope" formed by the family marks out a boundary with the outside world. It contains individual psyches. The boundary needs to be sufficiently permeable to allow exchanges—in some cases through processes of crisis—and avoid endogamous withdrawal. This forms our first dimension: The adolescent sees his or her family as being open to the outside [a].

As shown by Bion, the primary environment will convert the baby's emotions into thoughts [11]. The family provides a singular interpretation of the world which will leave its mark on the subject's identity, and will form a kind of marker linking the subject to his/her primary group. More generally, the child and later the adolescent will feel more or less supported by the family group. The second dimension retained is thus "the adolescent feels supported by his/her family" [b].

Adolescence revives conflicts of dependency and of desire for differentiation. It is a time that not only questions the adolescent's progress through life, but also raises different issues within the family, and in particular the conjugal affiliation bond. Adolescence questions the transforming effects of the family envelope. This angle of research enables an assessment of the quality of the boundaries between parents and children, which foster the distinction between the sensual and the affectionate. Thus the third dimension is "generational boundaries are marked out" [c].

Finally, each subject in the family group needs an identity and a place that are specific to him/her, this being true for the parental couple and for the different siblings. The fourth dimension is "each member of the family has his/her place" [d].

Thus the four question relating to the area of psychoanalysis are as follows:

- Does adolescent see his/her family as being open, to the outside?
- Does the adolescent feel supported by his/her family?
- Are generational boundaries marked out?
- Does each member of the family have his/her place?

Here too, the psychoanalyst rater is asked to indicate whether or not the adolescent's response provides information about the domain in question. If it does, the rater is then to allocate a score from 0 to 10 reflecting the degree of concordance between the situation described by the adolescent and the content of the item.

#### The clinical psychiatric perspective

According to the philosopher Georges Canguilhem, complaint and distress are the corner-stone of the concept of the “ill person” [12]. One of the main objectives of a physician during a clinical interview is therefore to listen, to characterize the patterns of distress of the human being before him/her. In the field of intrafamilial relationships, a child and adolescent psychiatrist will thus be keen to look for painful relationships between a patient and his/her parents or brothers and sisters. This is all the more important because epidemiological studies consistently show that family functioning can be a risk factor for psychiatric disorders, in particular in the area of suicide in adolescents [13]. It is also noteworthy that some patterns of family functioning can be resilience factors [14] and this is the reason why both negative and positive relationships with family are regularly looked for by clinicians. Consequently, we proposed four dimensions that assess the level of positive and negative relationships with parents and siblings. Two different series of dimensions were proposed for positive ([a] and [c]) and negative ([b] and [d]) relationships. This because it cannot be hypothesized that positive relationships are merely the opposite of negative relationships, especially in the period of adolescence when ambivalence and mixed emotions are especially frequent [15].

One important element that emerges from a clinical interview is the level of concern that the doctor feels towards his or her patient. It is from this feeling of concern that the doctor will decide simply to follow-up, to prescribe a treatment or to propose hospitalization. The emergence of concern of this sort is well explained by the conjunction of a semiological and a phenomenological approach. The semiological position relies on the observation of clear-cut and meaningful signs that are known to be important prognostic factors. For instance, a personal history of suicide attempt will be likely to play an important role in the decision to hospitalize a depressive patient. Conversely, the phenomenological position relies on the progressive emergence of a feeling of concern during the interview [16]. Our hypothesis is that the corpus that the adolescent provides about his/her family generally makes it possible for a clinician to appreciate his/her own level of concern regarding the adolescent. This is the object of dimension [e].

The five questions relating to the domain of clinical psychiatry are thus as follows:

- How does the adolescent perceive relationships with his/her parents or step-parents? (in reference to positive relationships, with a score from 0 for neutral to 10 for extremely positive)
- How does the adolescent perceive relationships with his/her parents or step-parents? (in reference to negative relationships, with a score from 0 for neutral to 10 for extremely negative)
- How does the adolescent perceive relationships with his/her siblings? (in reference to positive relationships, with a score from 0 for neutral to 10 for extremely positive)
- How does the adolescent perceive relationships with his/her siblings? (in reference to negative relationships, with a score from 0 for neutral to 10 for extremely negative)
- Is the rater concerned about the state of the patient?

In each case, the rating psychiatrist is asked to indicate whether or not the adolescent's response provides information on the domain in question. If it does, he/she is asked to allocate a score from 0 to 10 reflecting the level of concordance between the situation described by the adolescent and the content of the item.

#### The common dimension

Finally, the three groups of researchers independently considered that an adolescent's feeling of belonging to his/her family was relevant. The corresponding dimension [a] was therefore added and it was to be rated by all the raters, whether they were sociologists, psychoanalysts or child and adolescent psychiatrists:

[a] Does the adolescent feel he/she is part of the family? (the rater is to indicate whether or not the adolescent's answer provides information about the dimension. If the answer is yes, the rater is then to allocate a score from 0 to 10 for the strength of the feeling of belonging).

#### Improvement of rating reliability

Because we feared a low level of inter-rater reliability for the answers to the 18 questions presented above, for each item, we specified a typical situation corresponding to each possible answer (from 0 to 10). For instance, concerning the sociological item “Recognition of rights”, a score of 2 would correspond to the adolescent feeling controlled, expressed for instance by the words "my mother often checks on everything I do", while a score of 8 would correspond to the adolescent having a lot of freedom, or able to practice an activity he/she enjoys, with no mention of anything being forbidden, expressed in statements such as "I generally concentrate on enjoying myself, like going out with friends or doing activities I like".

### Statistical Analysis Plan

Descriptive statistics were based on barplots, percentages, means and standard deviations. To determine if the three sets of items shared a substantial common core, the statistical unidimensionality of all the 18 items was assessed (18 items corresponding to the 6 items designed by the sociologists, 4 by the psychoanalysists, 5 by the child and adolescent psychiatrists and the 3 items for “belonging”). A parallel analysis was conducted for this purpose since it appears as a method with optimal properties [17]. In case these 18 items might not be strictly unidimensional, factor analysis was used to determine potential independent latent factors (maximum likelihood estimator, varimax rotation). Interrater agreement was estimated with the weighted Kappa for the scores and for the Cohen Kappa for the occurrence of missing data for a given item.

Missing data were imputed using the median for the estimation of the eigenvalues required for the screeplot. Factor analyses were performed on the pairwise correlation matrix. Screeplots and factor analyses were estimated from the average of the scores obtained from the two raters from the same discipline. To assess robustness of results, we did a series of sensitivity analyses. All analyses were done successively: on a dataset with missing data imputed by the median, on a dataset with missing data imputed by a Gibbs sampler and on a dataset without imputation of missing data. In addition, factor analyses were done both with a promax or varimax rotation. Results being remarkably close, only the first series of analyses will be presented in the results section.

It has to be noticed that because no test of hypothesis is proposed in this paper, the clustered nature of data (multiple ratings per subject) has not to be taken into account in the statistical analyses.

All analyses were performed on R 3.0.0 software.

### Rights of Human Subjects

The study was approved by the “Comité consultatif sur le traitement de l'information en matière de recherche” (CCTIRS) and the “Commission Nationale Informatique et Liberté” (CNIL), with number MG/CP 10962. Informed consent was obtained from the parents and the participating adolescents. Adolescents gave a written informed consent collected during classroom. These written consents are stored in INSERM U669 research unit. All parents received a letter with an explanation of the objectives and content of the study and had the possibility to refuse the participation of their child. This procedure has been approved by CCTIRS and CNIL (national IRB for this type of study). Implicit and not explicit written consent was obtained from parents, for feasibility reasons and because this is a standard procedure for school surveys in France.

## Results

### Example of corpus provided by two adolescents

The average length of the 194 adolescents’ responses was 232 words, with a standard deviation of 129. Below are presented two examples of these answers.

The first one was obtained from a 13-ear-old boy:

"In my family me and my sister are always fighting, and our parents punish us because we're hitting each other, they have us do a hundred lines about not hitting. At mealtimes we talk, we watch the news. We go to bed at half past nine. If we bring bad marks home from school, with my parents we re-do the exercise so I can understand what I didn't get right. My sister is bright and top of the class, it's different from me because I'm nearly the bottom of mine. Sometimes my Mum and Dad quarrel. We eat a lot of meat. With potatoes and peas. At weekends we sometimes have fish, or go to a restaurant at lunchtime (but not always) and in the evening we all make our own sandwiches"

The second example comes from a 17-year-old girl:

"I have a pretty good relationship with my Mum, we're quite close, and if we get annoyed it's for small unimportant things. In the week she's away at work, so we see each other at weekends, which is not much, because with out-of-school activities the weekend is pretty full. So in the week I'm with my Dad and my big brother. My Dad isn't used to cooking, so we all do our own. My brother has a job, every other week he works mornings, and the other week he works afternoons and evenings, so we don't see each other much, and at the week he goes out. The household work isn't very well shared out, my Mum does most. I don't get on well with my Dad, we argue, but no more than that. I do get on with my brother so long as we don't get at each other too much. Meals are always good fun. My parents agree easily to me going out, and are ready to take me places and fetch me back, and my brother is too if it's a bit late for my parents. My parents don't go out much and I think it's a pity".

### Description of the study population

Among results in Table 1, we find a mean age of 14,7 years (sd. 2) and a proportion of 51% girls. Seventy-one percent of the adolescents were living with their two parents. There was an average number of 4.3 persons living in the same house and the average size of the sibship was 2 (s.d. 1.3). For about 27 percent of the adolescents in the sample, French and at least one other language were spoken at home.

**Table 1:** Description of the study population.

	Middle school	High school	TOTAL
**Gender**			
Boys	60.7 (n = 68)	38.5 (n = 32)	51.3% (n = 100)
Girls	39.3 (n = 44)	61.4 (n = 51)	48.7% (n = 95)
**Age**	13.14±1.02 (n = 110)	16.70±0.53 (n = 83)	14.66±1.96 (n = 193)
**You are living with**			
Both parents	63.4 (n = 71)	78.3 (n = 65)	69.7 (n = 136)
Your father	2.7 (n = 3)	4.8 (n = 4)	3.6 (n = 7)
Your mother	25.9 (n = 29)	12.1 (n = 10)	20.0 (n = 39)
Alternately father and mother	6.3 (n = 7)	3.6 (n = 3)	5.1 (n = 10)
Other situation	1.8 (n = 2)	1.2 (n = 1)	1.5 (n = 3)
**At home with your parents, what language do you speak**			
Only French	65.2 (n = 73)	81.9 (n = 68)	72.3 (n = 141)
French and another language	33.9 (n = 38)	18.1 (n = 15)	27.2 (n = 53)
**Number of siblings**	1.99±1.38 (n = 112)	1.98±1.29 (n = 83)	1.98±1.34 (n = 195)
**What is your father’s profession**			
Farmer	5.2 (n = 5)	0	2.8 (n = 5)
Self-employed	11.5 (n = 11)	7.4 (n = 6)	9.6 (n = 17)
Managerial	17.7 (n = 17)	43.2 (n = 35)	29.4 (n = 52)
Intermediate profession	18.8 (n = 18)	18.5 (n = 15)	18.6 (n = 33)
White collar worker	8.3 (n = 8)	9.9 (n = 8)	9.0 (n = 16)
Manual worker	35.4 (n = 34)	21.0 (n = 17)	28.8 (n = 51)
Retired	2.1 (n = 2)	0	1.1 (n = 2)

### Reliability of item scoring

The percentages of “0” ratings (which indicate a floor effect), the percentages of “10” ratings (ceiling effect) and the percentage of responses for which a given item was not assessable are presented in Table 2.

**Table 2:** Item analysis.

Discipline	Name of dimension dimension	Floor effect (% of 0)	Ceiling effect (% of 10)	Information not available (%)	Kappa for information available y/n	Kappa for score
Sociology	Belonging	0.01	0.03	0.02	0.74	0.62
Sociology	Affective environment	0.01	0.01	0.03	0.75	0.71
Sociology	Freedom	0.01	0.01	0.50	0.67	0.87
Sociology	Recognition	0.01	0.00	0.59	0.61	0.83
Sociology	Part of a whole	0.21	0.07	0.02	0.74	0.90
Sociology	Conflict	0.50	0.02	0.09	0.49	0.78
Sociology	Injustice	0.59	0.02	0.12	0.44	0.74
Psychoanalysis	Belonging	0.00	0.04	0.00	NA	0.70
Psychoanalysis	Openness	0.00	0.01	0.12	0.16	0.54
Psychoanalysis	Support	0.00	0.05	0.03	0.18	0.77
Psychoanalysis	Boundaries	0.00	0.02	0.02	0.33	0.33
Psychoanalysis	Place	0.00	0.03	0.03	0.16	0.52
Psychiatry	Belonging	0.01	0.02	0.02	NA	0.50
Psychiatry	Pos relations Parents	0.07	0.02	0.05	0.23	0.62
Psychiatry	Neg relations Parents	0.44	0.02	0.05	0.23	0.74
Psychiatry	Pos relations siblings	0.10	0.05	0.29	0.70	0.65
Psychiatry	Neg relations siblings	0.35	0.01	0.29	0.71	0.51
Psychiatry	Clinical opinion	0.72	0.00	0.03	NA	0.66
All	Belonging					0.44

Floor effect (percentage of lowest response), ceiling effect (percentage of highest response), percentage of responses concluding that a given item was not assessable, and inter rater agreement (Kappa coefficient).

Concerning the sociological items, responses relating to belonging and to the affective atmosphere provided a good amount of data with no floor or ceiling effect. Responses relating to freedom and recognition of personal merit were not very informative (small variance). Responses relating to the family forming a whole, to conflict and to injustice showed a floor effect (which was fairly moderate for the "forming a whole" effect). Concerning the psychoanalytic items, responses were fairly complete with neither floor nor ceiling effect. For the psychiatric items, there was a slight floor effect for the items concerning negative relationship with parents, negative relationship with siblings, and for the clinical assessment. The large number of missing responses should also be noted for the two items relating to siblings.

The Kappa coefficient was used to assess inter-rater reliability. According to Fleiss [18], kappas over .75 are considered as “excellent”, from .40 to .75 as “fair to good”, and below .40 as “poor”. In Table 2, all kappas related to sociological items are “fair to good” or “excellent”. Concerning the psychoanalytical and psychiatric items, except for the item about intergenerational boundaries (for which the inter-rater agreement was “poor”), all inter-rater agreements on the scores themselves are “fair to good” or “excellent”. Some inter-rater agreements about missing data for an item are only “poor”. For instance, concerning the item “negative relationships with siblings”, if no mention of this point was available in a text, rater 1 considered that it was not possible to rate the item while rater 2 considered that the rating was “0” (data not shown).

### Unidimensionality of the set of items

**Fig 1:**
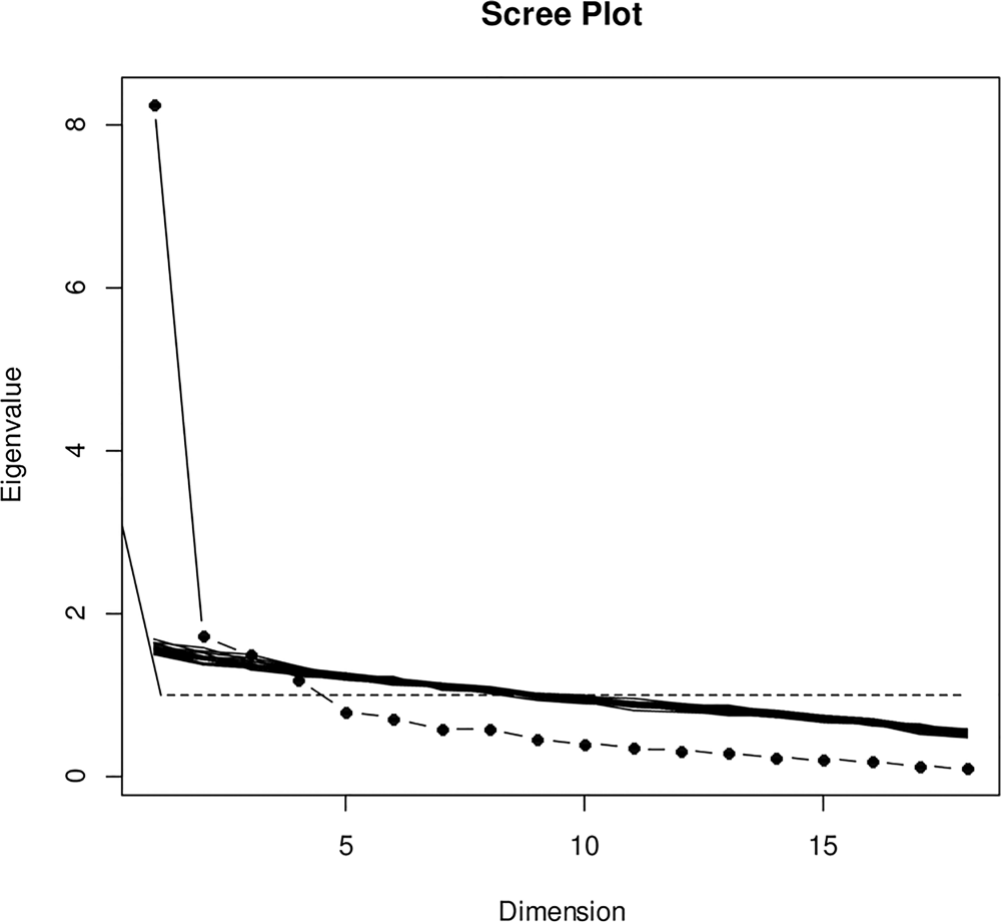
Screeplot. Screeplot of the eigen values for the item correlation matrix, parallel analysis. In addition to the scree plot of the study data, 20 random data sets with the same number of subjects and items were generated and the corresponding scree plot represented. This can be used to determine eigen values above “what could be expected by chance”.

The screeplot (Fig 1) is in favor of unidimensionality (the first eigenvalue is clearly above the others, it is the only value obviously above what could be expected by chance, and it represents 50.4% of the total variance). A solution with 2 factors can however also be envisaged because the second eigenvalue, even if it is negligible compared to the first, appears to be above that could be expected by chance in the the simulated screeplots. Finally, if one follows the classic but overly lax “Keiser rule” (eigenvalues above 1), a solution with 4 factors is of potential interest.

### Factor Analysis

As expected, the 1-factor model opposes items with negative feelings (negative relations with parents, negative relations with siblings, clinical concern, injustice, conflict) to the other items, all related to positive feelings.

Results of the 2-factor model reveal a first factor with high loadings on positive feelings in general and in particular with feelings of belonging (observed by the sociologists, psychoanalysts and psychiatrists), positive relationships with family (parents and siblings) and good emotional atmosphere. The second factor has high loadings on negative feelings (items “negative relationships with parents”, “conflicts” and “injustice”). Using an oblique rotation (promax), the correlation between these two factors can be estimated to be -0.64.

The first two factors of the 4-factor model correspond to the 2-factor solution. The 3^rd^ factor relates to individuation (it contrasts items which underline the singularity of each family member's “generational boundary”, “each member has a place”, with the item “parents as being part of a whole”). The 4^th^ factor covers the two items related to relationships with siblings.

## Discussion

Numerous family assessment measures already exist and our intention was not to develop and promote a new one. Instead, this paper aimed to look with a statistical perspective and across theoretical frameworks to the level of diversity in the manners intra-familial interactions are perceived by adolescents. More precisely, when disciplines as different as sociology, psychoanalysis and child and adolescent psychiatry are asked to characterize intra-familial interactions, do they catch authentic different facets or do they apprehend mostly the same dimension? The empirical study designed to answer this question used a mixed method design: an open question asked of adolescents and a rating of the answers collected on the basis of 18 dimensions related to 3 different theoretical frameworks.

The main result of this study is the existence of a strong underlying factor that explains a large amount of variance in all the 18 ratings coming from the 3 disciplines. This factor is bipolar, it reflects the level of positivity/negativity of the adolescent’s point of view concerning his/her intra-familial relationships. This result can however be refined. Beyond this single common factor two factors can be considered. One is related to positive feelings and the other to negative feelings. Of course, these two factors are highly, negatively correlated (r = -0.65). But there is here something that is well known by clinicians [15] and psychometricians [19]: negative feelings are not merely the opposite of positive feelings. Ambivalence is common, and this appears to be true in particular concerning intra-familial relationships as considered by adolescents. Two other factors can also be estimated, they are however at the very limits of statistical relevance. One concerns individuation and the other relationships with siblings.

Of course, these results need to be moderated because of obvious limitations in the study design. By construction, we are restricted here to intra-familial relationships as viewed by adolescents. These relationships are described in a short written text which can be sometimes insufficiently informative (as shown by a non-negligible percentage of missing answers to some items). In line with this methodological limitation, the theoretical frameworks proposed by the 3 teams of researchers had to be compatible with the elements available in the collected corpus. This perhaps led to a restriction in the level of complexity of the concepts that were tackled, which possibly induced an artificially simple factor structure for the dimensions assessed. Moreover, the statistical approach used here (i.e. unidimensionality conceived according to a psychometric standpoint) is not indifferent. The property of local independence which underpins the notion of psychometric unidimensionality does not equate with semantic equivalence. Local independence of a set of items corresponds to a situation where the responses to these items are independent conditional to a measured or unmeasured variable. The items have thus “only one thing in common” and the importance of this common core can be captured by the amount of variance explained by the common factor. Semantic equivalence is a notion that is much more subtle and delicate to define and to assess. Finally, the results have only a limited level of generalizability because of the sampling process, restricted to a given country at a particular moment. The sample is stratified according different categories of age and localization (rural versus urban), but obviously there is no ambition of representativeness there.

These weaknesses are however counterbalanced by authentic strengths. First, the size of the sample studied (close to 200) is substantial and compatible with the statistical routines that have been used [20]. In addition, all ratings were performed blind by two independent raters from each discipline. It can be noted that inter-rater reliability is globally good according to usual standards [18]. This was a real challenge, because the content of many items is subtle and because most raters were not used to quantitative evaluations. This is indeed encouraging as regards the feasibility of multi/inter-disciplinary research grouping together specialists from distinct epistemologies and using very different methodologies. One strength of the present study is the nature of data collected. A real bottom-up process, which stems from the qualitative research paradigm, guided the study. The dimensions were designed from the analysis of texts written by adolescents during a pilot study and the scores allocated for the pivotal study are derived from similar free texts. The ratings are thus obtained from the patient perspective itself and not from guided answers to oriented questions. The main strength of the paper comes surely from its clear-cut main result: a mostly dominant first principal component explains more than 50% of variance, while the second component explains less than 10% and is at the limit of “what could be expected by chance”. The structure of variance of the 18 items is thus compatible with the existence of a unidimensional common core. And this was not expected at the beginning of the study when we defined the three theoretical frameworks. One is based on recognition (being recognized by the other members of the family), one on the psychodynamic constitution of subjectivity and the last on the phenomenological and semiological perception of tension or distress. This means we have here three different ways of considering and gauging intra-familial relationships and, curiously, only one main common core ultimately remains.

Now, on a more conceptual viewpoint, it can be shocking to find only one dimension behind these three different and sophisticated theoretical frameworks. Indeed, how is it possible that social and cultural context (for the sociologists), inner life and development of psychic structures (for the analysts), and clinical symptoms and syndromes (for the psychiatrists) can be summarized without any substantial loss in a unique dimension (positive versus negative feelings about intra-familial relationships)? Does this mean that these theories are somewhat spurious, that they introduce elements of highbrow complexity where reality is in fact straightforward? Indeed this paper cannot have the pretention to deal with this issue. Our search for a common core is based on psychometric paradigm and we have to remember that statistical unidimensionality is not synonymous with semantic equivalence. Certain variables may account for only a small amount of the variance of a phenomenon while they are nevertheless of crucial importance to explain it. It would be in particular the case for a necessary condition that is ubiquitous: it explains 0% of variance of the phenomenon under study, but it is however indispensable to understand it. More important, this study relies on a four-stage reduction process that necessarily weakened the scope of its conclusions: 1/ by construction, the corpus analyzed is limited; 2/ the sociological, psychoanalytical and psychiatric perspectives are considered through a narrow and limited angle; 3/ they are reduced then to a list of 18 quantitative items; 4/ the covariance matrix of the responses to these items is reduced with the use of factor analysis. But reduction is inherent in all quantitative works. No doubt that some qualitative content analyses of the present corpus would cast a different light on the question of the existence of a common core. Obviously, this should be done and presented in another paper.

A final question has thus to be raised at this point. Considering the limitations presented above, are there any practical implications to the present study? Yes, and not only because this work proves that the complexity and diversity of family functioning is not necessarily a curse. There is some room for working together and for common constructs. Indeed, the main implication of this paper concerns evaluation studies. When performing a randomized controlled trial or a cohort study to evaluate a family therapy or a family intervention, there is a need for a primary endpoint, and preferably for a single main primary endpoint to preserve the type one error of the statistical test of hypothesis that will determine the conclusion of the trial [21]. If the phenomenon under study is highly multidimensional, it is difficult to propose a main primary endpoint that will satisfy all viewpoints and the conclusion of the trial will thus be open to criticism. This happens frequently in medicine, for instance in the evaluation of cancer treatments where a small improvement in survival can be criticized because it is obtained at the cost of a deterioration in quality of life. We have proved here that when considering the adolescent’s point of view concerning his/her intra-familial relationships, there is, formally, one single way to quantify the positivity/negativity of this viewpoint, whatever the theoretical framework used to structure it. This is a good news for all those who work for the evaluation and the promotion of family interventions.
